# Supplementary material for: Evaluation of the Effect of Pb Pollution on Avian Influenza Virus-Specific Antibody Production in Black-Headed Gulls (Chroicocephalus ridibundus)
Source: Animals (Basel). 2023 Jul 18;13(14):2338. doi: 10.3390/ani13142338 (PMC10376737; doi:10.3390/ani13142338)
Supplement: Supplementary file 1 [file animals-13-02338-s001.zip › Supplementary Table S1.pdf]

Supplementary Table S1. Virus strains used in neutralization tests.

| HA protein | Virus strains |
|---|---|
| H1 | A/swine/Iowa/15/1930 (H1N1) |
| H2 | A/Singapore/1/1957 (H2N2) |
| H3 | A/duck/Ukraine/1/1963 (H3N8) |
| H4 | A/duck/Czechoslovakia/1956 (H4N6) |
| H5 | A/duck/Hokkaido/Vac-3/2007 (H5N1) |
| H6 | A/duck/Hong Kong/960/1980 (H6N2) |
| H7 | A/seal/Massachusetts/1/1980 (H7N7) |
| H8 | A/turkey/Ontario/6118/1968 (H8N4) |
| H9 | A/duck/Hokkaido/49/1998 (H9N2) |
| H10 | A/chicken/Germany/N/1949 (H10n7) |
| H11 | A/duck/England/1/1956 (H11N6) |
| H12 | A/duck/Alberta/60/1976 (H12N5) |
| H13 | A/gull/Maryland/704/1977 (H13N6) |
| H14 | A/mallard/Astrakhan/263/1982 (H14N5) |
| H15 | A/duck/Australia/341/1983 (H15N8) |
| H16 | A/duck/Hokkaido/WZ82/2013 (H16N3) |
