# Supplementary material for: Evaluation of the Effect of Pb Pollution on Avian Influenza Virus-Specific Antibody Production in Black-Headed Gulls (Chroicocephalus ridibundus)
Source: Animals (Basel). 2023 Jul 18;13(14):2338. doi: 10.3390/ani13142338 (PMC10376737; doi:10.3390/ani13142338)
Supplement: Supplementary file 1 [file animals-13-02338-s001.zip › Supplementary Table S2.pdf]

Supplementary Table S2. The datasets analyzed for this study.

Sample number	Populations	Periods	Age	Sex	BW (g)	Pb (µg/dL)	Infection history	Antibody titer (OD)
1	TBP	Wi	Ad	F	297.0	2.00	N	0.16
2	TBP	Wi	Ad	F	242.0	0.67	N	0.54
3	TBP	Wi	Ad	F	250.0	1.09	P	0.81
4	TBP	Wi	Ad	F	274.0	4.03	N	0.40
5	TBP	Wi	Ad	M	324.0	0.42	N	0.23
6	TBP	Wi	Ad	F	220.0	0.71	P	1.28
7	TBP	Wi	Ad	M	273.0	12.05	N	0.36
8	TBP	Wi	Ad	F	268.0	4.00	P	0.75
9	TBP	Wi	Ad	F	261.0	0.50	P	1.07
10	TBP	Wi	Ad	F	273.0	0.31	P	1.37
11	TBP	Wi	Ad	F	236.5	3.34	P	0.81
12	TBP	Av	Ad	M	280.0	0.84	N	0.21
13	TBP	Wi	Ad	F	300.0	1.00	P	1.34
14	TBP	Wi	Ad	M	303.0	1.00	P	0.64
15	TBP	Wi	Ad	M	320.0	2.00	N	0.22
16	TBP	Wi	Ad	M	304.0	2.00	N	0.25
17	TBP	Wi	Ad	F	294.0	1.00	P	0.87
18	TBP	Wi	Ad	F	338.0	7.39	P	0.62
19	TBP	Wi	Ad	F	281.0	2.00	P	1.66
20	TBP	Wi	Ad	F	245.0	8.00	N	0.49
21	TBP	Av	Ad	M	281.0	2.00	N	0.12
22	TBP	Wi	Ad	F	302.6	2.00	P	0.99
23	TBP	Wi	Ad	M	288.0	1.00	P	1.28
24	TBP	Wi	Ad	M	316.3	2.56	N	0.46
25	TBP	Wi	Ad	M	335.0	21.23	P	0.67
26	TBP	Wi	Ad	M	315.0	3.08	N	0.47
27	TBP	Av	Ad	M	270.0	1.07	N	0.35
28	TBP	Av	Ad	F	275.0	0.55	P	1.63
29	TBP	Wi	Ad	M	291.0	1.00	P	1.40
30	TBP	Wi	Ad	F	365.5	2.00	P	0.67

31	TBP	Wi	Ad	F	278.5	8.67	P	0.99
32	TBP	Wi	Ad	M	245.7	1.00	P	0.62
33	TBP	Wi	Y	F	211.8	1.10	N	0.43
34	TBP	Wi	Ad	F	277.3	2.00	N	0.50
35	TBP	Wi	Ad	F	268.3	8.68	N	0.60
36	TBP	Wi	Ad	M	303.9	2.00	N	0.51
37	TBP	Wi	Ad	F	280.0	2.00	N	0.39
38	TBP	Wi	Ad	M	289.4	1.00	P	0.67
39	TBP	Av	Ad	M	289.0	0.27	N	0.40
40	TBP	Wi	Ad	M	255.0	4.97	N	0.39
41	TBP	Wi	Ad	F	281.0	0.33	P	1.41
42	TBP	Wi	Ad	F	190.0	1.43	P	1.89
43	TBP	Wi	Ad	M	296.0	0.85	P	1.50
44	TBP	Av	Ad	M	290.0	0.22	P	2.05
45	TBP	Wi	Ad	M	285.7	2.57	N	0.58
46	TBP	Wi	Ad	M	336.0	1.00	P	0.91
47	TBP	Wi	Ad	M	302.0	2.00	N	0.26
48	TBP	Av	Ad	M	276.0	0.42	N	0.33
49	TBP	Wi	Y	F	254.0	1.26	N	0.37
50	TBP	Wi	Ad	M	327.0	2.00	P	0.95
51	TBP	Wi	Ad	M	298.5	1.00	P	0.89
52	TBP	Wi	Ad	F	261.3	2.00	P	0.78
53	TBP	Wi	Ad	M	335.7	2.00	P	0.68
54	TBP	Wi	Ad	F	256.4	3.96	P	0.65
55	TBP	Wi	Ad	M	295.6	1.00	N	0.45
56	TBP	Wi	Ad	M	255.0	12.82	P	0.79
57	TBP	Wi	Ad	M	338.5	1.00	P	0.66
58	TBP	Wi	Ad	M	331.0	4.03	P	0.64
59	TBP	Wi	Ad	M	341.0	2.00	P	0.85
60	TBP	Wi	Ad	M	279.5	2.68	N	0.52
61	TBP	Wi	Ad	F	263.0	7.00	N	0.34
62	TBP	Wi	Ad	F	302.4	1.00	P	0.99
63	TBP	Wi	Ad	F	266.0	5.38	P	0.66

64	TBP	Wi	Ad	M	282.0	1.00	N	0.52
65	TBP	Wi	Ad	F	266.6	0.94	N	0.12
66	TBP	Wi	Ad	M	289.0	2.00	N	0.18
67	TBP	Wi	Ad	F	269.0	1.87	N	0.51
68	TBP	Wi	Ad	M	315.0	1.23	N	0.34
69	TBP	Av	Ad	F	286.0	1.08	N	0.12
70	TBP	Wi	Ad	F	302.0	26.05	N	0.54
71	TBP	Wi	Ad	M	310.0	2.51	P	0.58
72	TBP	Wi	Ad	M	309.0	2.00	N	0.19
73	TBP	Wi	Ad	F	278.0	10.72	N	0.39
74	TBP	Wi	Ad	F	256.0	2.00	P	0.39
75	TBP	Wi	Ad	F	262.0	8.91	P	0.63
76	TBP	Wi	Ad	F	287.0	8.83	N	0.20
77	TBP	Wi	Ad	F	320.0	2.31	P	1.64
78	TBP	Av	Ad	M	277.0	2.00	N	0.16
79	TBP	Wi	Ad	M	344.0	1.39	N	0.27
80	TBP	Wi	Ad	F	283.0	2.00	P	0.81
81	TBP	Wi	Ad	M	280.0	17.37	P	0.72
82	TBP	Wi	Ad	M	345.0	2.00	P	0.87
83	TBP	Wi	Ad	M	292.0	15.66	P	1.00
84	TBP	Av	Ad	M	295.0	0.28	N	0.31
85	TBP	Av	Ad	M	244.0	0.93	P	0.91
86	TBP	Av	Ad	F	263.0	2.00	P	0.93
87	TBP	Av	Ad	F	288.0	1.00	P	1.11
88	TBP	Av	Ad	F	273.0	0.57	N	0.16
89	TBP	Wi	Y	F	234.8	3.50	N	0.17
90	TBP	Av	Ad	F	227.0	0.60	N	0.51
91	TBP	Av	Ad	M	255.0	1.01	P	1.12
92	TBP	Wi	Ad	F	265.0	1.00	P	0.96
93	TBP	Wi	Ad	M	304.0	2.00	P	0.98
94	TBP	Wi	Ad	F	276.0	10.81	N	0.56
95	TBP	Wi	Ad	F	282.0	1.00	P	1.70
96	TBP	Wi	Ad	F	296.0	15.98	N	0.32

97	TBP	Wi	Ad	M	307.0	8.38	P	1.99
98	TBP	Wi	Ad	M	314.0	2.27	N	0.19
99	TBP	Wi	Y	F	292.0	0.80	N	0.51
100	TBP	Av	Ad	F	249.0	0.61	N	0.10
101	TBP	Wi	Ad	F	303.0	2.00	P	0.93
102	TBP	Wi	Ad	M	327.0	3.15	P	0.74
103	TBP	Wi	Ad	F	248.0	7.39	N	0.29
104	TBP	Av	Ad	M	301.0	0.24	N	0.27
105	TBP	Av	Ad	F	254.0	1.00	N	0.26
106	TBP	Av	Ad	F	280.0	0.74	N	0.41
107	TBP	Av	Ad	M	260.0	0.23	N	0.15
108	TBP	Wi	Ad	M	287.0	0.42	N	0.45
109	TBP	Av	Ad	F	276.0	1.00	P	1.44
110	TBP	Av	Ad	M	227.0	0.33	P	1.29
111	TBP	Av	Ad	M	278.0	0.18	N	0.30
112	TBP	Av	Ad	M	280.0	0.24	N	0.25
113	TBP	Av	Ad	F	275.0	0.40	N	0.22
114	TBP	Av	Ad	M	261.0	1.19	N	0.33
115	TBP	Av	Ad	M	278.0	8.86	P	0.54
116	TBP	Av	Ad	F	290.0	1.00	P	1.17
117	TBP	Av	Ad	F	272.0	0.26	N	0.29
118	TBP	Wi	Ad	M	329.0	0.53	P	1.76
119	MBP	Wi	Ad	F	276.9	12.62	N	0.33
120	MBP	Wi	Ad	F	301.0	3.50	N	0.43
121	MBP	Wi	Ad	F	222.1	4.99	P	0.75
122	MBP	Wi	Ad	M	288.0	2.00	P	1.08
123	MBP	Wi	Y	M	230.2	1.03	P	1.38
124	MBP	Wi	Ad	F	279.0	1.60	N	0.21
125	MBP	Wi	Y	M	274.8	3.00	P	0.69
126	MBP	Wi	Ad	F	263.0	1.70	P	0.74
127	MBP	Wi	Ad	F	276.0	4.60	P	0.61
128	MBP	Wi	Y	M	358.0	1.00	N	0.46
129	MBP	Wi	Ad	F	276.0	1.97	P	1.10

130	MBP	Wi	Ad	M	339.0	0.91	P	2.23
131	MBP	Av	Ad	M	313.0	1.30	N	0.52
132	MBP	Av	Ad	F	240.8	31.60	P	0.92
133	MBP	Av	Ad	M	282.0	4.60	N	0.43
134	MBP	Av	Ad	F	275.0	4.50	P	0.73
135	MBP	Wi	Ad	F	236.3	1.97	P	0.79
136	MBP	Wi	Ad	F	255.5	11.93	P	0.62
137	MBP	Wi	Ad	F	278.5	1.00	P	1.01
138	MBP	Wi	Ad	F	254.0	1.00	P	1.04
139	MBP	Wi	Ad	F	347.0	3.50	P	0.63
140	MBP	Wi	Ad	M	315.5	0.91	P	3.41
141	MBP	Wi	Ad	F	245.7	2.44	P	1.29
142	MBP	Wi	Ad	F	254.4	12.00	P	0.84
143	MBP	Av	Ad	F	214.6	1.00	P	0.78
144	MBP	Wi	Ad	M	292.0	28.90	P	0.81
145	MBP	Wi	Ad	F	258.6	2.00	P	1.29
146	MBP	Av	Ad	F	255.3	1.00	P	1.30
147	MBP	Wi	Ad	F	275.0	1.00	P	1.16
148	MBP	Wi	Ad	F	278.8	1.50	P	1.02
149	MBP	Wi	Ad	F	247.6	5.52	P	1.32
150	MBP	Wi	Ad	M	303.5	1.00	P	1.12
151	MBP	Av	Ad	F	317.0	1.00	P	1.66
152	MBP	Wi	Ad	M	311.2	6.66	P	0.88
153	MBP	Wi	Ad	F	261.9	1.00	P	1.02
154	MBP	Av	Ad	F	253.6	6.20	P	0.70
155	MBP	Wi	Ad	F	268.1	2.73	P	1.86
156	MBP	Wi	Ad	F	233.0	1.00	P	1.19
157	MBP	Wi	Ad	M	334.0	4.80	P	1.33
158	MBP	Wi	Ad	M	296.0	2.00	P	0.96
159	MBP	Wi	Ad	F	294.0	1.00	P	1.13
160	MBP	Wi	Ad	F	290.5	1.90	N	0.47
161	MBP	Wi	Y	M	358.0	2.38	P	1.34
162	MBP	Wi	Y	M	280.0	6.52	P	1.10

163	MBP	Wi	Y	M	236.0	8.00	P	1.54
164	MBP	Av	Y	F	299.0	0.48	P	1.27
165	MBP	Wi	Ad	F	279.2	2.00	N	0.27
166	MBP	Wi	Ad	F	256.0	1.00	P	1.84
167	MBP	Av	Ad	F	267.5	1.00	P	2.67
168	MBP	Av	Ad	M	274.5	2.00	P	1.61
169	MBP	Wi	Ad	F	286.0	13.10	P	0.67
170	MBP	Wi	Ad	F	284.5	7.90	P	1.77

Annotation: populations (Tokyo Bay population: TBP, Mikawa Bay population: MBP), periods (Arriving: Av, Wintering: Wi), age (Yearling: Y, Adult: Ad), sex (Male: M, Female: F), BW: body weight, BLL: blood Pb level, and infection history (negative: N, positive: P), OD: optical density.
